# The Feasibility and Potential Impact of Brain Training Games on Cognitive and Emotional Functioning in Middle-Aged Adults

**DOI:** 10.1089/g4h.2017.0032

**Published:** 2018-02-01

**Authors:** Paula M. McLaughlin, Ashley F. Curtis, Laura M. Branscombe-Caird, Janna K. Comrie, Susan J.E. Murtha

**Affiliations:** ^1^ Ontario Neurodegenerative Disease Research Initiative (ONDRI), Western University, London, Canada.; ^2^ York University, Department of Psychology, Toronto, Canada.

**Keywords:** Brain training, Aging, Executive cognition, Anxiety

## Abstract

***Objectives:***To investigate whether a commercially available brain training program is feasible to use with a middle-aged population and has a potential impact on cognition and emotional well-being (proof of concept).

***Method:*** Fourteen participants (ages 46–55) completed two 6-week training conditions using a crossover (counterbalanced) design: (1) experimental brain training condition and (2) active control “find answers to trivia questions online” condition. A comprehensive neurocognitive battery and a self-report measure of depression and anxiety were administered at baseline (first time point, before training) and after completing each training condition (second time point at 6 weeks, and third time point at 12 weeks). Cognitive composite scores were calculated for participants at each time point.

***Results:*** Study completion and protocol adherence demonstrated good feasibility of this brain training protocol in healthy middle-aged adults. Exploratory analyses suggested that brain training was associated with neurocognitive improvements related to executive attention, as well as improvements in mood.

***Conclusion:*** Overall, our findings suggest that brain training programs are feasible in middle-aged cohorts. We propose that brain training games may be linked to improvements in executive attention and affect by promoting cognitive self-efficacy in middle-aged adults.

## Introduction

Over the past decade, commercially available brain training games have targeted middle-aged and older audiences with implicit and explicit claims of preventing or remediating age-related cognitive decline through “exercising your brain” techniques. These software platforms allege to support the “use it or lose it” hypothesis,^1,2^ where engaging in cognitively stimulating activities can attenuate or prevent age-related changes in cognition.

In contrast to the well-documented cognitive benefits of action-based videogames in younger cohorts,^3^ the impact of brain training games on cognitive aging is less clear. This genre of games uses components from traditional psychological measures (e.g., mental arithmetic and pattern recognition) to train underlying cognitive functions associated with the tasks (e.g., working memory and perceptual reasoning, respectively). These games typically use feedback and reinforcement to promote learning, with task difficulty manipulated to facilitate within game skill acquisition—a critical feature for skill transfer.^1^

To date, the research shows inconsistent findings on the effectiveness of the brain training programs in the aged. Some studies have demonstrated improvements in controlled processing, attention, memory, and executive skills using commercially available games (*Nintendo Brain Age*,^4^ *Lumosity*,^5–8^ *Nintendo DS Brain Training*^9^) and custom-designed platforms,^10,11^ with cognitive gains related to the participants' perceived cognitive abilities and quality of life.^9^ In contrast, other studies have reported no cognitive gains in older participants^12^ and no transfer effects to untrained tasks in individuals aged 18–60 years.^13^ These inconsistent findings may reflect differences in participant demographics, training programs (within-subject, crossover vs. between-subject design), control conditions (mentally stimulating vs. no-training/no contact), and/or outcome measures (standardized neuropsychological tests vs. experimental computerized tasks).

Interestingly, these studies have primarily focused on individuals older than 60 years, despite cognitive decline beginning decades earlier.^14^ The dearth of intervention studies on middle-aged cohorts is likely related to recruitment and engagement issues. That is, middle-aged participants are difficult to recruit and fully engage in intervention studies because they are usually working full-time and/or have other competing commitments. As such, although commercially available games are marketed toward this demographic group, it is unclear whether brain training programs aimed at improving cognition can actually be effectively applied to middle-aged populations, where decline is first noted. To understand the necessary parameters and practicality of this intervention approach to cognitive aging, a feasibility study is necessary. In the present study, we investigated the feasibility of using a commercially available brain training game (Nintendo DS *Big Brain Academy*) as an interventional tool that could improve neurocognitive functioning in middle-aged adults.

As depression and anxiety are known to negatively impact cognition, are related to early cognitive decline,^15,16^ and have been associated with increased risk of dementia,^17^ we also investigated the influence of brain training on emotional functioning. To date, there is limited research exploring the effect of brain training on mood and anxiety. Preliminary findings suggest that brain training games can attenuate depressive symptoms in healthy older adults.^18^ Likewise, other gaming platforms that integrate play with physical activity (e.g., Nintendo^®^ *Wii™ Sports* or *Fit*) have been shown to reduce depressive symptoms in older adults^19^ and stroke patients,^20^ as well as symptoms of anxiety and depression in Lupus patients.^21^ Given the impact of mood and anxiety on cognition and well-being, it is important to identify ways to mitigate these symptoms early in the aging process. Therefore, in addition to our cognitive indices, we examined symptoms of anxiety and depression as outcome measures in our participants.

In sum, we investigated the feasibility of using a commercially available brain training game as a potential interventional tool to improve neurocognitive and emotional functioning in an understudied group that is critically relevant to cognitive aging (i.e., middle-aged adults).

## Materials and Methods

### Participants

Fourteen middle-aged adults (ages 46–55), recruited from the community surrounding York University in Toronto, Ontario, participated in the study. Participants were entered into a draw for a Nintendo DS for participating. All participants provided informed consent, were fluent in English, and were nongamers (i.e., reported playing less than 1 hour of video or brain training games per week over last 2 years). Exclusion criteria included the following: history of neurological or psychiatric illness, head injury, or alcohol/substance abuse; presence of an untreated sleep disorder; and taking medication(s) known to impact cognition (e.g., antipsychotics, psychotropics, and benzodiazepines).

### Design and procedure

The study was approved by the York University Research Ethics Board and conducted in accordance with the Helsinki Declaration. Using a crossover design, participants completed two 6-week training conditions: experimental brain training game playing (GP) condition and active control answering trivia questions (TQ) condition. The training condition order was counterbalanced. Participants were required to complete 18 one-hour training sessions (3 hours/week) per condition at their homes, and were asked to keep a journal documenting their hours and performance. Participants were contacted weekly to verify adherence to the training.

#### GP condition

Participants were provided with a Nintendo DS gaming system and *Big Brain Academy* software. *Big Brain Academy* consists of 15 activities grouped into five categories (think, memorize, analyze, compute, and identify) designed to stimulate/train and practice mental abilities. Activities required various cognitive skills, including simple attention and working memory, perceptual reasoning, and visuospatial skills (see Table 1 for a description of the expected skills associated with each activity^12^). All activities had a speeded component and increased in difficulty as the player advanced through the game. During the training sessions, participants could practice a specific activity or complete a test that consisted of five randomly selected activities (one from each category). Each activity took 60 seconds to complete and players were provided with trial-by-trial accuracy feedback. In addition, a performance score was given at the end of each activity in terms of “grams” of brain weight, with higher values reflecting better performance. On completing a test, players were also provided with an overall performance score (sum of the five activity scores), letter grade, and information on what areas needed improvement. Participants were asked to document and submit their weekly highest test score (“brain weight”) across the 6 weeks of training.

**Table 1.** Nintendo DS Big Brain Academy Categories, Activities, and Proposed Underlying Abilities

*Categories and activities*	*Underlying ability*
Think	Perceptual reasoning
Heavyweight	Quantitative and analogical reasoning
Pathfinder	Nonverbal spatial reasoning
Bone yard	Nonverbal spatial reasoning
Memorize	Memory
Memo-random	Visual working memory
Sound bites	Nonverbal auditory attention
Flash memory	Visual working memory
Analyze	Visuospatial perception
Animal lines	Perceptual speed, scanning
Missing link	Spatial integration
Cube count	Visuospatial perception
Compute	Mathematics and working memory
Coin-parison	Numerical estimation
Add agency	Numerical computation
Written math	Numerical computation
Identify	Object and spatial perception
Shadow shift	Visual inspection (dynamic)
Get in shape	Spatial integration
Matchmaker	Visual matching

#### TQ condition

Participants answered a set of randomly generated TQ on a variety of topics, including music, history, science, art, and literature (e.g., “How fast is hypersonic?”). Participants were required to use the Internet during this condition to control for the amount of time spent interacting with technology. They were allowed to use any available online resource to answer these questions. Explicit feedback was not provided during this condition, and task difficulty was not manipulated.

#### Outcome measures

Study completion and protocol adherence (self-reported and submitted weekly test scores) rates were used to assess feasibility. A neuropsychological testing battery, administered at baseline (before training) and after completing each training condition, provided measures of neurocognitive (i.e., “near” skill transfer) and emotional change. The battery included measures of attention and processing speed, memory, and executive skills (see Table 2 for measures). Participants were also administered the Hospital Anxiety and Depression Scale (HADS)^22^ at each time point to measure emotional functioning. This 14-item self-report questionnaire assesses the presence and severity of anxiety (e.g., worrying thoughts, restlessness) and depression (e.g., anhedonia, apathy) symptoms with solid validity (Cronbach's α = 0.67–0.93).^23^ Finally, general intellectual functioning^24^ was measured at baseline. Each testing session took ∼90 minutes to complete and alternate forms were used whenever possible.

**Table 2.** Demographic Variables and Neuropsychological Test Scores Across Training Order Groups

	*GP-TQ training order*			*TQ-GP training order*		
*Variable*	*Baseline*	*Game training*	*Trivia questions*	*Baseline*	*Trivia questions*	*Game training*
Age (years)	52.7 (2.4)	—	—	52.1 (3.4)	—	—
Education (years)	14.1 (1.2)	—	—	13.3 (1.5)	—	—
Sex (M:F)	2:5	—	—	3:4	—	—
WASI: FSIQ-2	112.4 (7.3)	—	—	113.3 (9.4)	—	—
Executive attention composite	223.9 (14.6)	242.3 (17.4)	232.1 (18.1)	212.6 (16.1)	211.9 (28.2)	222.0 (25.1)
TMT-A	53.1 (5.0)	57.1 (7.5)	54.7 (4.2)	51.6 (6.5)	50.9 (8.6)	51.4 (13.6)
TMT-B	55.9 (6.1)	63.1 (5.4)	58.4 (10.3)	52.3 (12.9)	55.1 (16.5)	57.6 (9.1)
WASI: matrix reasoning	59.4 (4.2)	60.1 (5.3)	61.7 (4.3)	59.3 (3.5)	55.9 (9.8)	59.1 (5.7)
Letter (FAS) fluency	55.4 (14.4)	61.9 (14.4)	57.3 (12.0)	49.4 (8.8)	50.0 (6.1)	53.9 (10.7)
Verbal memory composite	71.9 (14.1)	80.43 (16.4)	87.6 (14.7)	76.1 (9.3)	83.9 (9.4)	88.7 (20.6)
HVLT—immediate	37.0 (8.3)	41.9 (7.6)	44.1 (7.0)	40.6 (5.6)	43.4 (5.7)	45.3 (11.6)
HVLT—delayed	34.9 (8.2)	38.6 (10.0)	43.4 (8.7)	35.6 (7.9)	40.4 (6.7)	43.3 (9.5)
Nonverbal associative learning and memory composite	130.9 (15.3)	109.7 (8.5)	126.6 (22.0)	119.9 (10.9)	115.0 (15.1)	124.3 (16.1)
BVMT—immediate	47.4 (7.6)	36.9 (6.0)	43.7 (10.9)	40.4 (9.4)	37.4 (7.6)	40.1 (9.2)
BVMT—delayed	45.1 (9.6)	36.4 (4.7)	44.4 (10.1)	42.9 (3.9)	40.4 (9.3)	47.1 (9.3)
WAIS: digit symbol copy	38.3 (5.3)	36.4 (4.4)	38.4 (6.4)	36.6 (4.7)	37.1 (4.8)	37.0 (6.3)
Attention/language composite	99.3 (17.0)	111.3 (25.3)	103.3 (13.6)	102.7 (10.0)	103.6 (12.0)	103.4 (13.8)
WAIS: digit span total	55.4 (7.8)	55.9 (8.2)	57.0 (4.9)	54.7 (8.5)	54.6 (8.0)	57.4 (8.0)
Category (animal) fluency	43.9 (14.4)	55.4 (20.1)	46.3 (10.2)	48.0 (6.0)	49.0 (9.0)	46.0 (7.1)
Emotional functioning						
HADS-anxiety	6.6 (2.9)	3.0 (0.8)	3.6 (2.2)	6.4 (2.4)	5.4 (2.2)	3.4 (2.1)
HADS-depression	1.7 (2.1)	1.3 (1.8)	2.1 (2.3)	3.4 (2.2)	3.1 (2.1)	2.4 (2.6)

All neuropsychological test data represent standardized T scores (education and/or age corrected), with composite scores reflecting the sum of T scores associated with that factor.

BVMT, Brief Visuospatial Memory Test-Revised (forms 2, 3, and 4); HADS, Hospital Anxiety and Depression Scale (raw score out of 21); GP, game playing; HVLT, Hopkins Verbal Learning Test-Revised (forms 2, 3, 4); TMT, Trail Making Test (versions 1 and 2); TQ, trivia questions; WAIS, Wechsler Adult Intelligence Scale-Third Edition; WASI: FSIQ-2, estimated full-scale IQ score, two-subtest form Wechsler Abbreviated Scale of Intelligence.

### Data preparation

Based on data collected simultaneously in our laboratory for another study,^25^ we conducted an initial exploratory factor analysis to determine the structure of the neurocognitive battery. A principal component factor analysis with an Oblimin rotation and Kaiser Normalization was completed on the standardized T scores (based on education- and/or age-adjusted norms) obtained from 91 healthy community dwelling adults (ages 18–82; including the baseline data from the 14 individuals who participated in the present study), with no reported history of neurological or psychiatric illness, head injury, and/or alcohol/substance abuse. A four-component solution best explained the data, with 70.3% of the variance accounted for using this structure (Table 3). Based on this four-factor solution, composite scores (sum of T scores) were calculated for participants in the present study at each time interval. These composite scores were labeled as follows: Executive attention (Trail Making Test—Parts A and B^26^; Matrix Reasoning^24^; letter fluency^26^); Verbal memory (Hopkins Verbal Learning Test-Revised^27^: immediate and delayed recall); Nonverbal associative learning and memory (Brief Visuospatial Memory Test-Revised^28^: immediate and delayed recall; Digit Symbol Coding^29^); and Attention/language (Digit Span^29^; category fluency^26^).

**Table 3.** Summary of the Demographic Variables, Neuropsychological Test Scores, and *r* Coefficients from the Principal Component Factor Analysis

*Variable*	r	*Mean*	*SD*	*Range*
Age		45.30	19.53	18–82
Education		14.57	2.21	10–21
Sex (F:M)		58:33		
HADS-anxiety		6.07	3.28	0–14
HADS-depression		3.00	2.62	0–11
Factor 1: nonverbal associative learning and memory				
BVMT—delayed	0.906	50.86	10.07	20–67
BVMT—immediate	0.838	50.05	10.63	20–69
WAIS: digit symbol copy	0.644	56.13	12.21	30–80
Factor 2: executive attention				
TMT-A	0.791	51.35	9.59	20–70
TMT-B	0.732	52.71	11.78	13–81
WASI: matrix reasoning	0.542	56.65	10.02	23–74
Letter (FAS) fluency	0.509	49.81	9.97	29–77
Factor 3: attention/language				
WAIS: digit span total	0.884	52.80	9.50	33–80
Category (animal) fluency	0.643	49.14	9.98	28–72
Factor 4: verbal memory				
HVLT—delayed	0.902	44.35	10.18	21–63
HVLT—immediate	0.893	42.93	9.76	20–66

All neuropsychological test data represent standardized T scores (education and/or age corrected).

## Results

Demographic data and mean performances across measures are presented in Table 2. Training order groups were well matched on demographic variables, estimated intellectual functioning, and baseline measures (*P* > 0.10).

### Outcome measures

#### Protocol feasibility

All participants successfully completed the study, including both training conditions and three outcome assessments. Across weekly check-ins, all participants reported completing the training protocol as required (i.e., three 1-hour training sessions/week) in both conditions (self-reported adherence = 100%). During the GP condition, participants documented and submitted their weekly highest *Big Brain Academy* test score on the majority of the training weeks (86.9%). Of the 14 participants, eight documented and submitted their test scores each week (57%).

#### Neurocognitive and emotional

Proportional changes in neurocognitive performance and anxiety and depression scores following the experimental GP condition relative to baseline are presented in Table 4 for each participant. In addition, proportional improvements on *Big Brain Academy* test scores after 6 weeks of training (relative to the first week) are provided. Within task skill acquisition (test scores) and “near” skill transfer was generally observed. The majority of participants showed improvements in executive attention (86%), attention/language (79%), verbal memory (71%), and *Big Brain Academy* test score (79%). The majority of participants also reported fewer symptoms of anxiety (93%). In addition, just under half of the participants reported fewer symptoms of depression (43%). No participants reported more symptoms of anxiety or depression following the GP condition. In contrast to these favorable findings, only 36% showed an improvement in nonverbal associative learning and memory, with *declines* noted in most participants (64%).

**Table 4.** Proportional Changes (%) in Neurocognitive Performance, Anxiety, and Depression Following the Experimental Game Playing Brain Training Condition Relative to Baseline for Each Participant

*Participant*	*Nonverbal associative learning and memory*	*Executive attention*	*Attention/language*	*Verbal memory*	*HADS-anxiety*	*HADS-depression*	*Big Brain Academy*
1	−22.86	16.89	−13.89	29.73	−33.33	−50.00	2.45
2	−13.22	6.37	27.40	18.18	−57.14	−100.00	19.12
3	−16.89	16.59	16.13	12.12	−75.00	0.00	25.37
4	−6.78	−1.28	2.99	27.06	−20.00	0.00	25.20
5	−31.13	0.82	31.34	−1.14	−50.00	0.00	12.09
6	−20.80	8.70	−13.25	3.00	−40.00	0.00	5.47
7	5.31	10.63	47.92	−6.59	−66.67	−16.67	100.00
8	14.81	−7.65	8.97	1.10	0.00	−50.00	−5.14
9	−3.60	0.98	25.00	9.26	−40.00	−50.00	−15.74
10	−2.44	15.54	22.47	−11.54	−85.71	−100.00	15.88
11	5.15	0.45	2.41	−7.21	−33.33	0.00	112.86
12	5.00	7.98	−27.78	2.94	−57.14	0.00	25.37
13	11.45	10.27	46.84	8.70	−50.00	0.00	−0.56
14	−5.45	2.83	40.00	1.14	−55.56	0.00	68.96
*Mean*	−5.82	6.37	15.47	6.20	−47.42	−26.19	27.95
*SD*	13.69	7.36	23.37	12.30	22.14	37.39	38.78

Proportional changes on neurocognitive and emotional functioning measures are based on the following equations: GP-TQ group: (scores at six-weeks) − (scores at baseline)/(scores at baseline) × 100; TQ-GP group: (scores at 12-weeks) − (scores at baseline)/(scores at baseline) × 100. For proportional changes in *Big Brain Academy* test scores, the following equation was used: (score at six-weeks) − (score at one-week)/(score at one-week) × 100. Positive values on the neurocognitive measures represent improvements in performance. Whereas negative values on the HADS represent reductions in reported symptoms.

When examining the proportional changes following the control TQ condition relative to baseline (Table 5), a similar pattern of results was observed. That is, the majority of participants performed better on measures of executive attention (64%), attention/language (79%), and verbal memory (57%). In addition, most participants reported fewer symptoms of anxiety (79%), with 21% endorsing fewer symptoms of depression. Again, similar to the experimental GP condition, only 43% showed an improvement in nonverbal associative learning and memory, with *declines* in performance noted in 57% of the participants.

**Table 5.** Proportional Changes (%) in Neurocognitive Performance, Anxiety, and Depression Following the Control Trivia Questions Condition Relative to Baseline for Each Participant

*Participant*	*Nonverbal associative learning and memory*	*Executive attention*	*Attention/language*	*Verbal memory*	*HADS-anxiety*	*HADS-depression*
1	−13.57	0.46	15.28	−17.12	−100.00	50.00
2	7.44	7.35	38.36	12.50	−28.57	0.00
3	10.81	10.92	12.90	−1.52	−37.50	0.00
4	22.88	−0.85	10.45	29.41	0.00	0.00
5	−34.44	6.97	0.00	1.14	−33.33	0.00
6	−11.20	−5.65	20.48	1.00	−80.00	0.00
7	2.65	7.25	72.92	12.09	−58.33	16.67
8	3.70	−10.71	8.97	−7.69	33.33	0.00
9	−15.32	−10.73	0.00	0.00	−40.00	−50.00
10	−20.33	1.55	4.49	−7.69	−14.29	−40.00
11	−6.62	5.00	−7.23	−7.21	−11.11	50.00
12	−5.83	1.26	13.89	8.82	−42.86	20.00
13	−0.76	9.38	25.32	6.09	50.00	0.00
14	19.09	0.00	31.67	14.77	−33.33	−14.29
Mean	−2.96	1.58	17.68	3.19	−28.29	2.31
SD	15.64	6.90	20.25	11.85	39.64	27.61

Proportional changes on neurocognitive and emotional functioning measures are based on the following equations: GP-TQ group: (scores at 12-weeks) − (scores at baseline)/(scores at baseline) × 100; TQ-GP group: (scores at six-weeks) − (scores at baseline)/(scores at baseline) × 100. Positive values on the neurocognitive measures represent improvements in performance. Whereas negative values on the HADS represent reductions in reported symptoms.

To explore these training effects further, we compared the proportional changes associated with the GP condition with the changes associated with the control TQ condition using a series of paired sample *t*-tests. Overall, participants showed greater improvements in executive attention following the GP condition relative to the TQ condition, *t*(13) = 2.52, *P* = 0.026. Likewise, participants showed a greater reduction in depressive symptoms following the GP condition relative to the TQ condition, *t*(13) = −2.79, *P* = 0.015. A similar nonsignificant pattern was observed with the anxiety index, with participants generally reporting fewer symptoms following the GP condition relative to the TQ condition, *t*(13) = −1.77, *P* = 0.100. In contrast, the proportional changes associated with verbal memory [*t*(13) = 0.74, *P* = 0.475], nonverbal associative learning and memory [*t*(13) = −0.63, *P* = 0.540], and attention/language [*t*(13) = −0.36, *P* = 0.728] were not significantly different between the conditions.

As suggested by the data presented in Tables 4 and 5, there appears to be a potential relationship between improvements in executive attention and reductions in anxiety associated with the experimental GP condition. To explore this potential association, we correlated the proportional changes on our neurocognitive measures (with Bonferroni control, *P* < 0.01) with the proportional changes on the HADS across training conditions. A significant relationship between improved executive attention and reductions in anxiety was found following the GP condition, *r* = −0.723, *P* = 0.003, but not following the control TQ condition, *r* = 0.047, *P* = 0.872. This suggests that as anxiety decreased on the GP condition, performance on executive attention tasks improved. No other correlations were found for either training condition (Table 6).

**Table 6.** Pearson Correlation Coefficients (*r*) Between the Proportional Changes (%) in Neurocognitive Performance and HADS Anxiety and Depression Scores for Each Training Condition

	*Experimental GP condition*		*Control TQ condition*	
*Variable*	r	P	r	P
HADS-anxiety				
Nonverbal associative learning and memory	0.182	0.533	0.264	0.361
Executive attention	−0.723^*^	0.003	0.047	0.872
Attention/language	−0.345	0.228	−0.198	0.497
Verbal memory	0.340	0.235	0.199	0.495
HADS-depression				
Nonverbal associative learning and memory	−0.011	0.970	0.057	0.846
Executive attention	−0.153	0.603	0.340	0.234
Attention/language	−0.098	0.738	0.095	0.746
Verbal memory	−0.048	0.871	−0.199	0.496

^*^ Significant correlation between proportional changes in neurocognitive performance and HADS score (*P* < 0.01).

In sum, our findings demonstrate good feasibility, with preliminary results showing promising efficacy. That is, improvements in neurocognitive performance and emotional well-being were observed following the experimental GP condition. Relative to our active control condition, engaging in the brain training program was associated with greater improvements on measures of executive attention, as well as reduced symptoms of depression. Interestingly, improvements in executive attention were associated with reductions in anxiety, but only for the experimental GP condition.

## Discussion

It is well established that select cognitive abilities decline with age, with measurable changes starting in middle age.^14,30^ Over the past several decades, researchers have been exploring different ways to ameliorate age-related decrements in cognition with varying success.^31^ Rarely, however, have studies reported on the impact of brain training on emotional functioning. Furthermore, the majority of programs have focused on older cohorts (ages 60+). In the present study, we explored the feasibility of using a commercially available brain training program as an intervention tool, as well as report preliminary findings on the impact of brain training on neurocognitive and emotional functioning in middle-aged adults. Study completion and protocol adherence demonstrated good feasibility of a brain training protocol in healthy middle-aged adults. In addition, preliminary findings suggest potential training efficacy (proof of concept), with target “near” skill transfer and improved affect observed. Specifically, participants showed a greater improvement in executive attention and a reduction in depressive symptoms following 18 hours of brain training over a 6-week period relative to an active control condition. Although participants also demonstrated improvements in attention/language and verbal memory relative to baseline performance, this effect was similar across training conditions and thus most likely reflects a practice effect. Interestingly, we found that proportional improvements in executive attention and reductions in anxiety were related following the experimental GP condition, although reductions in depressive symptoms were not associated with improvements in neurocognitive performance. As anxiety is known to interfere with attention and executive control,^16^ our results suggest that improved executive attention following our brain training intervention may be the consequence of reduced anxiety.

As suggested elsewhere,^32^ middle-aged adults commonly worry about cognitive changes and neurodegenerative conditions such as Alzheimer's disease. Consistent with previous findings of self-efficacy influencing cognitive training outcomes,^9^ we suggest that our brain training program may have promoted cognitive self-efficacy, which subsequently reduced anxiety in our cohort and resulted in performance improving on executive attention measures. That is, participants were able to engage in a cognitively stimulating activity, see their performance improve with practice (as measured by “brain weight”), and have a better understanding of their ability to enhance their cognitive skills after completing the brain training condition. This likely resulted in reductions in anxiety and subsequent improvements in executive attention performance. Similar to most brain training games, *Big Brain Academy* provides constant feedback and task difficulty is titrated to the player's performance (with difficulty increasing as the player advances through the game and skills are adequately acquired). According to Green and Bavelier,^1^ this type of training procedure is likely associated with motivation and arousal. Therefore, by engaging in this brain training game program, it is possible that symptoms of anxiety were reduced (and cognitive performance subsequently improved) because participants were provided with feedback about their performance, were adequately challenged during training, and were able to observe improvements with practice.

In support of this interpretation, the initial improvements seen in the participants who completed the brain training condition first were diminished after completing the control condition (see GP-TQ group performance in Table 2). Specifically, executive attention and attention/language performance returned to baseline levels, with symptoms of depression and anxiety (at least partially) rebounding when participants were not engaged in the brain training condition. Future studies will need to directly measure cognitive self-efficacy to validate this interpretation. In addition, we recommend that the seemingly transient nature of the gains reported here be explored further. That is, future studies should determine whether individuals need to continually engage in brain training games to reap the suggested benefits, or can gains in cognition and affect be sustained with or without booster game training sessions. Demonstration of a lasting impact is particularly important when determining the utility of this cognitive aging intervention approach.

The relatively limited improvement in cognition associated with our brain training program is consistent with other studies that use similar crossover designs and standardized neuropsychological measures.^12^ It is possible that the “unspecified” nature of our training program may have interfered with showing more widespread cognitive gains. That is, we did not prescribe the *type* of brain training that needed to be completed (specific activities or tests on *Big Brain Academy*); rather, participants were allowed to freely engage with the game during the required hours of play and it was only requested that training be completed at the same time of day throughout the study. Although this maximizes the ecological validity of this training approach, future research should explore whether more structured programs are necessary to improve cognitive skills (i.e., provide prescribed training, while controlling for potential time-of-day effects). Furthermore, measuring the impact of brain-GP on cognition using more sensitive indices (e.g., computer-based tasks) instead of standard clinical measures would be beneficial.

The present study's design allowed us to measure the feasibility of brain training in middle-aged adults, as well as present preliminary outcome findings on neurocognitive functioning and affect. Although we successfully demonstrated the feasibility of using a commercially available brain training program as an intervention tool, our sample size limits our ability to generalize our conclusions on the impact of brain training in middle-aged populations. In addition, we only explored “near” skill transfer effects^*^—again limiting the application of these preliminary findings.

Contrary to previous brain training studies^5–8,10,11^ that use a no-contact/no-training control group, we included a cognitively stimulating active control condition and implemented a crossover design. This approach is generally considered superior in terms of scientific rigor, as it attempts to control for placebo effects while allowing participants to serve as their own control. However, a potential criticism of our design is that training conditions were not matched adequately on expectation of improvement or task characteristics.^33^ In general, matching expectations is a significant challenge in brain training game research, and findings such as ours may simply reflect a placebo effect. This genre of videogame is marketed to improve brain health and/or cognition, with explicit messages embedded within the game itself. This software design is not unique to *Big Brain Academy* and is found throughout commercially available brain training games. As such, finding an adequate control condition that can equate the expectation factor will be an important consideration moving forward.

Regarding task characteristics, brain training games use a complex learning paradigm that utilizes feedback and reinforcement to promote learning, with task difficulty appropriately manipulated to facilitate skill acquisition. These characteristics, which are challenging to control, are likely critical for skill transfer (as suggested elsewhere^1^). Again, future studies should attempt to match task characteristics across experimental and control training conditions.

In conclusion, our results demonstrate the feasibility of brain training in middle-aged adults using a commercially available game. Our novel, although preliminary, findings suggest a relationship between engaging in brain training games, reductions in anxiety, and improved executive attention. Indeed, improvements in executive attention appear to be related to reduced anxiety, possibly through a cognitive self-efficacy mechanism. To understand the mechanism of change proposed here, future studies will need to incorporate appropriate control condition(s) that consider task characteristics and potential placebo effects. In addition, it will be important for future studies to examine the transient nature of improvements found in the present study. Incorporating long-term follow-ups beyond the initial post-test period will be important. These methodological considerations (appropriate control conditions, with longer follow-ups) are surprisingly rare, but necessary to determine the extent to which reductions in anxiety may be linked to the brain training programs.

## Notes

^*^ A criticism of using neuropsychological tasks to measure outcome in brain training studies is that this genre of game incorporates elements of standard psychological measures. Therefore, it is argued that the participant is being trained on the outcome measures. Although there are some general similarities between training and outcome measures in the present study, there are also significant differences. These include how the test/training is completed (paper-and-pencil with a test administrator vs. independently with a gaming console), testing characteristics (auditory vs. visual presentation), and how performance is measured (accuracy vs. reaction time). Furthermore, there is only partial overlap between the *Big Brain Academy* activities and the neurocognitive battery in terms of underlying cognitive skills. For example, our outcome measures included tasks of verbal and nonverbal learning and recall, language, and word generation, which are not associated with any of the *Big Brain Academy* activities. Conversely, the outcome measures did not include mental arithmetic, visual working memory, or object and spatial perception measures–all of which are found in the training activities (Table 1).
